# Efficacy and predictors of success on laryngomalacia surgery: experience from a tertiary pediatric care center in Brazil

**DOI:** 10.1016/j.bjorl.2023.101315

**Published:** 2023-08-30

**Authors:** Renata Loss Drummond, Rita Carolina Pozzer Krumenauer Padoin, Bárbara Duarte Salgueiro, José Faibes Lubianca Neto

**Affiliations:** a Santa Casa de Misericórdia de Porto Alegre (UFCSPA), Serviço de Otorrinolaringologia e do Programa de Residência Médica em Otorrinolaringologia, Porto Alegre, RS, Brazil; b Hospital da Criança Santo Antônio, Serviço de Otorrinolaringologia Pediátrica, Programa de Fellowship em Otorrinolaringologia Pediátrica, Porto Alegre, RS, Brazil; c Universidade Federal de Ciências da Saúde de Porto Alegre (UFCSPA), Disciplina de Otorrinolaringologia (ORL) e Programa de Pós-Graduação em Pediatria, Porto Alegre, RS, Brazil

**Keywords:** Laryngomalacia, Stridor, Airway obstruction

## Abstract

**Objectives:**

Laryngomalacia is the most common congenital cause of stridor; the natural history of the disease runs through to complete resolution by the age of two. Severe cases are characterized by cyanosis, hypoxia, apnea, furcular and/or subcostal retractions, aspirations, pulmonary hypertension, and failure to thrive and must undergo surgery. This study aimed to evaluate the success rates of supraglottoplasty in our hospital and evaluate the predictive factors for surgical success.

**Methods:**

Cohort study, prospectively planned. 75 patients undergoing endoscopic surgery from July 2007 to July 2016 were analyzed at the Santo Antônio Children’s Hospital. The primary outcome was percentage of surgical success, defined as the absence of respiratory symptoms or presence of a mild stridor without retractions on the first post-operative month (late success). The secondary outcomes were the early surgical success (absence of respiratory symptoms or presence of a mild stridor without retractions on the first post-operative day).

**Results:**

39 (58.2%) were male, with an average of 4.9 months. Surgical success on the first day was 80.6% (*n* = 54). At the end of the 1st month, surgical success was 88.6%, considering only those who completed assessment. Twenty-one (34%) presented comorbidities. Presence of comorbidities, pharyngomalacia and GERD were associated with a worse result on the 1st postoperative day, whereas, at the end of the first month, presence of comorbidities, concomitant injuries (tracheo and bronchomalacia) and pharyngomalacia were the predictive variables of surgery failure.

**Conclusion:**

Supraglottoplasty has high rates of efficacy and low morbidity. The presence of comorbidities and pharyngomalacia has shown association with a worse early and late surgical outcome. Synchronous airway lesions predict a worse surgical result at the end of the first month. GERD was associated with obstructive symptomatology only in the 1st post-operative day.

**Level of evidence:**

Level 3 of evidence, according to the “The Oxford 2011 Levels of Evidence” from Oxford Centre for Evidence-Based Medicine.

## Highlights

• A sample of 75 children with severe laryngomalacia undergone supraglottoplasty.
• The global success rate of the surgery on the first month was 77%.
• It was associated with low morbidity: no complications were found.
• Comorbidities and synchronous airway lesions reduced the surgical success rate.
• Gastroesophageal reflux was not shown to be a predictor of a poor surgical result.

## Introduction

Laryngomalacia is a cyclic inspiratory supraglottic collapse,^1^, ^2^ which can lead to different intensities of respiratory obstruction. Current theories attribute this collapse to predisposing anatomical alterations and, primarily, to alterations of the laryngeal neuromuscular tonus.^3^, ^4^, ^5^ It is the most common congenital cause of stridor, responsible for up to 75% of cases.^6^, ^7^, ^8^

Clinically, it manifests as inspiratory stridor most commonly perceived between the second and sixth week of life,^9^ which worsens during feeding, agitation, crying, and in the supine position. The natural history of the disease involves the worsening of symptoms between four and eight months, followed by an improvement between eight and twelve months and complete resolution by two years of age.^6^

Laryngomalacia can be divided into mild, moderate, and severe, based on clinical findings. The mild form covers cases in which the stridor is not accompanied by increased respiratory effort or by interruptions while eating. Patients with moderate disease present self-limiting intermittent dyspnea, which creates greater difficulty eating, involving increased mealtimes, suffocating, coughs, and vomiting. Severe cases, in turn, are characterized by cyanosis, hypoxia, furcular and/or subcostal retractions, aspirations, pulmonary hypertension (in some cases even *cor pulmonale*), and delayed weight and height gain.^4^, ^10^, ^5^ Independently of the intensity, laryngomalacia significantly alters the quality of life of those affected.^11^

It is estimated that, in around 60% of infants, laryngomalacia is associated with Gastroesophageal Reflux Disease (GERD).^12^ The causal relationship, however, has not yet been established.^13^ It is speculated that GERD fosters the contact of acid with supraglottic structures, which would lead to edema and desensitization of the protective laryngeal reflex.^3^ For that reason, it is recommended that patients with laryngomalacia and eating difficulties are treated for GERD with acid suppression therapy.^14^

Both clinical and surgical treatments significantly improve patients’ quality of life.^15^ Currently, it is known that a surgical indication is reserved for severe cases, which represent fewer than 20% of those diagnosed.^16^ The standard procedure is supraglottoplasty.^17^ Its rate of success in improving breathing and eating varies from 38.1% to 100%,^18^ with recurrences and post-operative complications being rare.^6^, ^16^ The association with other comorbidities (neurological, heart, or genetic diseases), present in up to 45% of cases, configures the greatest predictor of surgical failure.^5^, ^19^, ^20^, ^21^, ^22^

The aim of this study is to evaluate the success rate in the first month post-surgery of children who underwent supraglottoplasty in our hospital in the period from July of 2007 to July of 2016. Secondarily, the predictive factors of surgical success were evaluated.

## Methods

A prospectively planned cohort study was conducted. It initially included patients with severe laryngomalacia undergoing any surgical technique via endoscopy in the period from July of 2007 to July of 2016, at the Santo Antônio Children’s Hospital. The initial sample was composed of 75 cases. It did not include patients with late onset laryngomalacia, defined as two years of age or older children with new diagnosis of laryngomalacia and no previous history of stridor.

The study was approved by the Research Ethics Committee of the institution (opinion 5.695.661) and all participants’ parents signed informed consent.

For the laryngomalacia diagnosis, the following clinical variables were evaluated: onset of stridor, worsening and improving factors, perinatal antecedents, the presence of other congenital anomalies. Those with significant commorbities or severe cases (difficulty eating, low weight gain, episodes of cyanosis and apnea or complications that required hospitalization due to respiratory obstruction), underwent video nasal-pharyngeal-laryngeal bronchoscopy in a surgical center using a Machida® 3.2 mm diameter endoscope under surface inhalational general anesthesia in spontaneous ventilation, inspecting the airway until the level of the secondary bronchi branch. Next, rigid laryngeal-tracheal bronchoscopy was carried out with a Karl-Storz® 4 mm diameter endoscope in search of potential secondary lesions. All examinations carried out were recorded.

The criteria for surgical indication were severe laryngomalacia with a) Clinical evidence of severe respiratory obstruction, manifested by cyanotic crises with the presence or not of subcostal and intercostal retractions and/or pectus excavatum and/or b) Feeding disorders with stunted weight and height development.

The patients were evaluated in relation to the presence of associated comorbidities and the presence of GERD. The definition of GERD was based on clinical and laryngoscopic criteria (hyperemia of the arytenoids and posterior pachydermia). No patient underwent impedance-pHmetry.

The surgeries were carried out under inhalational (sevoflurane) and endovenous (propofol and fentanyl) general anesthesia, under spontaneous ventilation, in all cases without the use of orotracheal intubation, using only a catheter with nasotracheal oxygen.

The surgery was carried out with cold instruments, in an individualized manner, based on the anatomical-functional alterations present. In the case of short aryepiglottic folds, these were excised with micro scissors.^24^ In the case of associated redundant mucosa of the arytenoids, supraglottoplasty was carried out, which involved the resection of the aryepiglottic folds, of the mucous redundancy, and, if necessary, of the lateral surface of the epiglottis. In the case of inspiratory epiglottic retroversion, glossoepiglottopexy was chosen, with cauterization of the lingual surface of the epiglottis and of the base of the tongue, with or without suturation.

In the immediate post-operative phase, the patients were sent to the Pediatric Intensive Care Unit (PICU), where they remained for 24 h; treatment with anti-reflexive therapy and corticotherapy was started immediately, in all patients. The criteria for hospital discharge were the establishment of a secure oral feeding, without the need for supplementary oxygen therapy and with mild or no effort under spontaneous ventilation. The follow-up was carried out on an outpatient basis.

The primary outcome was percentage of surgical success, defined as the absence of respiratory symptoms or presence of a mild stridor without retractions on the first post-operative month (late success). The secondary outcomes were the early surgical success (absence of respiratory symptoms or presence of a mild stridor without retractions on the first post-operative day).

The data analysis was conducted using the PASW statistical package version 19 and with a Microsoft Excel 2010 electronic spreadsheet. All the tests were run assuming a 5% alpha error. Frequency (%) and mean (IQR) measures were used and after verifying data normality, the Mann Whitney *U* test was run for non-normal continuous variables, as well as the Chi-Square test to compare proportions.

## Results

Of the initial sample of 75 participants, eight were excluded from the study using the following criteria: three were aged over 30 months at the time of the diagnosis; three underwent the glossoepiglottopexy technique, a less frequent surgical technique; and two needed another complementary surgical procedure (mandibular distraction).

It was observed that 54 children (80.6%) were asymptomatic and/or had mild symptoms already on the first day. There was a follow-up loss of 14 patients (12 were from distant towns and did not return for the one-month assessment and two patients could not be contacted) and, at the end of the first month, 47 (77%) presented no respiratory symptoms. The 6 children (9.0%) who had severe stridor or required tracheostomy after 30 days and the 14 (20.9%) cases that lost follow-up were considered as surgical failures.

In the surgical failure group, 70% were females, 85% had more than one type of laryngomalacia, 55% had concomitant airway lesions (of which 55% were pharyngomalacia, 10% tracheomalacia, and 5% supraglottic hemangioma). 90% underwent only aryepiglottic fold resection. 40% had associated comorbidities. The 6 patients who remained with severe stridor or respiratory effort after 30 days underwent tracheostomy.

Of the 67 patients studied, 39 (58.2%) were of the male sex, and their ages varied from five days to 30 months, presenting a mean of 4.9 months. Among the children included in the study, GERD was found in 26 patients (42%). Of the individuals considered, 21 (34%) presented comorbidities; among these, the most common were craniomaxillofacial anomalies, found in six patients (10%); followed by neurological ones, which in isolation were found in 9% of the patients. In 12%, the neurological ones were combined with other comorbidities. Cardiovascular ones were presented in isolation in 3% of the patients and in 6% they were associated with other comorbidities. Among the concomitant airway lesions, pharyngomalacia, tracheomalacia, and bronchomalacia were considered, either in isolation or association; pharyngomalacia was analyzed separately as it was the most prevalent of the synchronous lesions. 25 patients (41%) presented other concomitant airway alterations, pharyngomalacia being the most common (35.9%). The clinical variables and outcomes can be seen on Table 1. No complications were reported (supraglottic stenosis, granuloma formation, or clinically evident aspiration).

**Table 1:** Clinical variables and outcomes^a^.

	Assintomatic 1st day, *n* (%)	Significance (*p*)	Assintomatic 1st month, *n* (%)	Significance (*p*)
Comorbities				
Present	14 (66)	<0.001	14 (70)	0.001
Absent	40 (100)		33 (100)	
Synchronous airway lesions^b^				
Present	19 (76)	0.11	16 (76.1)	0.02
Absent	35 (97.2)		31 (96)	
Pharyngomalacia				
Present	16 (72.7)	0.004	13 (72)	0.007
Absent	38 (97.4)		34 (97.4)	
GERD				
Present	20 (76.9)	0.014	20 (80)	0.06
Absent	34 (97.1)		27 (96.4)	

a *n* = 67 on the first postoperative day and *n* = 53 at the end of the first month.

b Amongst the synchronous airway lesions, pharyngomalacia was analyzed separately as it was the most prevalent one.

## Discussion

In 1922, Igauer described the first surgical procedure in the supraglottic larynx for correcting laryngomalacia, which was a partial resection of the epiglottis.^17^ In 1984, Lane et al. excised the lateral portions of the epiglottis and corniculate cartilages, reporting an important improvement of the laryngomalacia in a three-month-old infant.^23^ In 1985, Seid et al. described the excision of the aryepiglottic folds,^24^ followed by the work of Zalzal et al. in 1987, who demonstrated the efficacy of a similar procedure in 10 patients, consolidating the endoscopic supraglottoplasty technique.^25^ In the national literature, there are few studies and with little casuistry reporting the experience of tertiary hospitals with supraglottoplasty for severe laryngomalacia.^26^, ^27^, ^28^

Despite the definition of surgical success varying between different studies, improvement of the respiratory pattern and of eating are generally considered to be the post-operative outcomes; the success rates in the literature vary from 53% to 95%.^2^, ^8^, ^16^, ^25^, ^29^ An objective evaluation of surgical success is not yet well established, since some consider the persistence of mild stridor as failure and others consider moving from a state of severe stridor to mild stridor, without the patient’s everyday activities being compromised, as surgical success. It is believed that new studies should be conducted to standardize the results.^30^ The global success rate at the end of the first month was 77% of the initially evaluated patients; however, when the losses were excluded, clinical improvement occurred in 88.6% of the patients who completed the 30 day evaluation.

Regarding the use of the cold technique, studies suggest that there is no difference in the results in patients subjected to the procedure with laser or with cold instrumentation.^13^, ^31^ Some, however, relate the use of laser with an increase in complications from thermal injury to the tissues. As in the literature,^14^ most of the patients on our sample presented shortened aryepiglottic folds (63% or 94.02%), being isolated (22% or 32.8%) or associated with other supraglottic alterations.

A review by Zalzal et al. in 2012 showed that supraglottoplasty failures were due to one of two factors: insufficient supraglottic resection and/or patient comorbidities.^19^ Post-operative complications are rare, with rates lower than 10%, where aspiration is the most common long-term complication. The risk of post-operative aspiration is low and is generally observed in patients that already presented pre-operative symptoms.^29^ In the present investigation, no aspiration complications were found. Other complications include granuloma formation and supraglottic stenosis, both even rarer. These complications did also not occur in the present case study.

In relation to predictive factors of surgical success, it was demonstrated that the presence of comorbidities increases failure rates.^30^ Zalzal et al. concluded that children with neurological disease, heart disease, craniomaxillofacial malformation, and severe gastroesophageal reflux disease present a higher risk of surgical failure, needing corrective surgeries, a tracheostomy, or gastrostomy.^19^ Various studies have demonstrated that the presence of comorbidities is statistically associated with surgical failure;^28^, ^32^, ^33^ some authors even believe that surgical failures should warn of the possibility of underlying neurological disease.^22^ A meta-analysis by Preciado and collaborators demonstrated an approximately seven-fold increase in the risk of supraglottoplasty failure in the presence of comorbidities.^14^ However, the mechanism through which these patients present a worse surgical result is unknown. Multi-factor causes are believed to be responsible, such as hypotonia, cardio-pulmonary alterations, laryngeal edema, and respiratory effort. Children subjected to the procedure at less than two months old present a greater chance of surgical failure.^20^ Escher et al. related hypotonia, present in various neurological syndromes, to a worse post-operative result and highlighted that, in the presence of severe pharyngomalacia, supraglottoplasty in isolation may be inefficient.^32^ Another study observed that premature patients present worse results and that findings associated with shortened aryepiglottic folds and an epiglottic retroversion are related to the presence of other airway alterations.^34^ Durvasula et al. demonstrated that prematurity in isolation is not the cause of post-operative failure, but rather other associated comorbidities in these children.^33^

Douglas and collaborators demonstrated that patients that presented surgical failure are more prone to a diagnosis of neurological disease in the post-operative phase.^22^ In the study of Schroeder et al., 55% of patients with neurological disease evolved to post-supraglottoplasty tracheostomy, and the rest presented the need for ventilatory support and an increase in hospitalization time.^35^ Patients with cerebral ischemia (with cerebral palsy) had worse surgical results, even when compared to patients with other neurological diseases (27% vs. 4% success).^33^ In that same study, the success rate of syndromic patients (without neurological deficit) was worse, with micrognathia being associated with a worse prognosis, generally requiring other procedures (e.g., mandibular distraction, glossoepiglottopexy) to avoid a tracheostomy. In the present investigation, it was observed that, among the patients with comorbidities, 19 (70%) were symptomatic in the first month post-surgery, while 100% of the healthy patients were asymptomatic or mildly symptomatic (*p* =  0.001).

Synchronous airway lesions are present in 7.5%–64% of patients with laryngomalacia.^5^ In our study, that prevalence was 40.3%. It was shown that pharyngomalacia was a predictor of surgical failure, since when it was present, in isolation or combined with other comorbidities, only 27% of the children were asymptomatic in the first month, while in those not affected, that rate was 72% (*p* = 0.007, see Table 1). The “concomitant lesion” variable in our study was a compilation of cases of tracheomalacia and bronchomalacia associated with laryngomalacia. It was not associated with a worse early surgical prognosis, but it was shown to be the predictor of a poor surgical result at the end of the first month. Besides the effect of the loss of patients on that result, it is not discarded that some of these cases may have been prematurely undervalued, due to the presence of mild laryngomalacia, with the importance lying in the genesis of the symptoms of patients only recognized later, with the resolution of the laryngomalacia and permanence of the symptoms.

The presence of GERD is considered by some authors as universal in these patients.^14^ The prevalence of clinical GERD in the present study was 42%, a little lower than the prevalence in the literature, which lies between 65% and 100% of cases.^2^, ^36^, ^37^ An association was found between the presence of GERD and symptomatology in the first day post-surgery; however, although there is a tendency for failure in patients with GERD at the end of the first month, there was no statistically significant association (*p* =  0.06, Table 1). There are two possible explanations: either the loss of cases after 30 days, reducing the statistical power of the study; or, perhaps, the establishment of anti-reflux treatment in the post-operative phase, attenuating the effects of the GERD on the laryngeal mucous. One of the limitations of the present investigation was the lack of standardization for diagnosing GERD, which was based solely on the clinical standard and on the laryngeal endoscopy findings, without carrying out impedance pHmetry. This shortcoming, however, is also found in most of the studies that evaluate the results of supraglottoplasty.^3^

## Conclusion

It is concluded that supraglottoplasty is a surgery with high efficacy rates and associated with low morbidity. The association between laryngomalacia and other variables, such as the presence of comorbidities, synchronous airway lesions, pharyngomalacia, and GERD, can reduce the surgical success rate. It is believed that a better understanding of the pathophysiology of laryngomalacia and the reasons for failures in the supraglottoplasty can help in better future patient management.

## Funding

This research did not receive any specific grant from funding agencies in the public, commercial, or not-for-profit sectors.

## Conflicts of interest

There is no actual or potential conflict of interest including any financial, personal, or other relationships with other people or organizations within three years of beginning the submitted work that could inappropriately influence, or be perceived to influence, their work.
